# Back-Splicing Transcript Isoforms (Circular RNAs) Affect Biologically Relevant Pathways and Offer an Additional Layer of Information to Stratify NMIBC Patients

**DOI:** 10.3389/fonc.2020.00812

**Published:** 2020-05-22

**Authors:** Anshita Goel, Douglas G. Ward, Naheema S. Gordon, Ben Abbotts, Maurice P. Zeegers, K. K. Cheng, Nicholas D. James, Richard T. Bryan, Roland Arnold

**Affiliations:** ^1^Institute of Cancer and Genomic Sciences, College of Medical and Dental Sciences, University of Birmingham, Birmingham, United Kingdom; ^2^NUTRIM School for Nutrition and Translational Research in Metabolism & CAPHRI Care and Public Health Research Institute, Maastricht University, Maastricht, Netherlands; ^3^Institute of Applied Health Research, College of Medical and Dental Sciences, University of Birmingham, Birmingham, United Kingdom

**Keywords:** circular RNA, back-splicing, bladder cancer, tumor grade, NMIBC, biomarkers

## Abstract

Circularized transcript isoforms due to back-splicing are increasingly being reported in different tissues types and pathological states including cancer. Since these circular RNAs (circRNAs) are more stable than linear messenger RNA their identification and profiling in tumor tissue could aid in stratifying patients and may serve as biomarkers. In this study, we have investigated the relationship between circRNA expression and tumor grade in a cohort of 58, mostly non-muscle-invasive bladder cancer patients. From 4571 circRNAs detected, we identified 157 that were significantly differentially expressed between tumor grades relative to the linear transcript. We demonstrated that such grade-related differences can be identified in an independent cohort, and that a large fraction of circRNAs can be, in principle, detected in urine. The differentially expressed circRNAs cluster into subgroups according to their co-expression, subgroups which are enriched for DNA repair, cell cycle and intracellular signaling genes. Since one proposed function of circRNAs is to interfere with gene-regulation by acting as microRNA “sponges,” candidates which were differentially expressed between tumor grades were investigated for potential miRNA target sites. By investigating the circRNAs from bladder cancer related pathways we demonstrated that the expression of these pathways, the circRNAs, and their parental genes are often decoupled and do not correlate, yet that some circRNAs do not follow this tendency. The present study provides the next step for the comprehensive evaluation of this novel class of RNAs in the context of non-muscle-invasive bladder cancer. Intriguingly, despite their possible function as microRNA sponges, they potentially affect host mRNA levels at the transcriptional stage, as compared to post-transcriptional control by miRNAs. Our analysis indicates differences of their activity between bladder cancer tumor stages, and their relative expression levels may provide an additional layer of information for patient stratification.

## Introduction

Circular RNAs (circRNAs) are a class of single-stranded closed RNA molecules, created by backsplicing events from linear mRNA (1). They were first identified ~20 years ago, but were initially seen as artifacts of aberrant RNA splicing (2). However, more recently, numerous potential functions of circRNAs have been proposed, such as acting as miRNA sponges, modulating transcription and interacting with RNA-binding proteins (RBPs) (3). Additionally, several genome-wide studies indicate that circRNAs are evolutionary conserved across species (4). Furthermore, an increasing number of studies show that circRNAs are strongly correlated with the proliferation, apoptosis, invasion, and metastasis of human tumors, which indicates the potential of circRNAs to act as novel therapeutic targets and biomarkers (5). The use of circRNAs for the latter is especially intriguing since they are more stable than linear RNA forms, escaping degradation by those processes which are dependent on the recognition of the ends of RNA molecules.

With the help of novel bioinformatics approaches and appropriate sequencing methods, comprehensive studies of circRNA species are possible (6), allowing investigation of the landscape of circRNAs in healthy and cancerous tissues. circRNAs are generally expressed at low levels and often exhibit cell type specific and tissue specific patterns (7). Most circRNAs originate from exons of protein-coding genes and can consist of one or multiple exons; some circRNAs also arise from introns, intergenic regions, non-coding RNA (ncRNA) loci, and other portions of the genome (8). In eukaryotes, the lengths of circRNAs are heterogeneous, ranging from ~100 to 4000 base pairs (9), and are estimated to account for as many as 0.1–10% of molecules in the transcriptome (10).

Bladder cancer (BC) is the 12th most common cancer worldwide (6th most common in males) with 430,000 new cases reported in 2012 (11). Over three-quarters of new cases are diagnosed as non-muscle-invasive bladder cancer (NMIBC: stages Ta/T1/Tis), with the remainder demonstrating invasion of tumor into the detrusor muscle (muscle-invasive bladder cancer, MIBC: stages T2+) (12). MIBC is a life-threatening disease requiring radical treatment and carrying a poor prognosis (13). Although NMIBC is not immediately life-threatening, recurrence after initial treatment is common and 10–15% of cases will progress to MIBC, necessitating burdensome and expensive long-term surveillance (14, 15). NMIBC is thus a heterogeneous disease with regard to both clinical outcomes (recurrence and progression) and underlying biology, with considerable differences in chromosomal alterations and mutational events between grades (low/high grade or grades 1–3) and stages (Ta/T1/Tis) (16). Furthermore, expression analyses derived from largescale RNA sequencing initiatives have tended to classify NMIBCs into three subtypes: the Lund Group describe urobasal A (UroA), genomically unstable (GU), and infiltrated, overlapping with the UROMOL classification of Class 1 (UroA, -progression signature, -CIS signature), Class 2 (GU, +progression signature, +CIS signature), and Class 3 (pronounced expression of lincRNAs, decreased expression of genes associated with cell cycle and metabolic processes, and increased expression of genes associate with histone modifications and chromatin remodeling) (17).

More recently, the integration of chromosomal and expression alterations with the mutational landscape has identified six molecular subtypes of BC with different molecular features, prognoses and distributions between NMIBC and MIBC (18): the Neural-like subtype is prevalent in MIBC and characterized by high WNT/β-catenin signaling; HER2-like is distributed evenly across NMIBC and MIBC, with higher *ERBB2* amplification and signaling; Papillary-like is a NMIBC subtype enriched in urothelial differentiation genes with a high frequency of actionable *FGFR3* mutations, amplifications, and *FGFR3-TACC3* fusion; Luminal-like is also predominantly NMIBC, has higher MAPK signaling and more *KRAS* and *KMT2C/D* mutations than other subtypes; Mesenchymal-like and Squamous-cell carcinoma-like are predominant in MIBC. Importantly, about 20% of NMIBCs show MIBC subtype traits and a lower 5-yr OS rate than Papillary-like NMIBC (81 vs. 96%) (18).

Genomic and epigenomic approaches may thus inform the development of more accurate risk stratifiers and non-invasive diagnostics (19), tools that are urgently required by healthcare professionals and patients alike (20). CircRNAs also appear to have a role to play in this already complex setting as a novel class of prognostic biomarkers for NMIBC—in a pioneering study, Okholm et al. (21) evaluated circRNAs in a large cohort (*n* = 457) of NMIBCs and identified circRNAs that are differently expressed between high and low risk tumors, highlighting two (circHIPK3 and circCDYL) as potential biomarkers (21).

In this study, we investigate circRNAs in a set of 58 tumors and evaluate circRNAs, demonstrating that for a subset, their relative expression is tumor grade dependent and may represent an alternative or additional molecular classification. We also provide an initial analysis of the overlap of the circRNAs detected in our study with the results of Okholm et al. in order to evaluate reproducibility between different tumor sample sets and alternative computational pipelines.

## Materials and Methods

### Samples and Library Preparation for RNA-Sequencing

Fresh-frozen tumors were collected as part of the West Midlands' Bladder Cancer Prognosis Programme (BCPP). This study was carried out in accordance with the recommendations of the International Committee on Harmonization Good Clinical Practice (ICH GCP) guidelines. The protocol was approved by the NHS Health Research Authority East Midlands—Derby Research Ethics Committee (ref: 06/MRE04/65). All participants gave written informed consent for the donation of tissue biospecimens and their subsequent utilization in biomedical research. RNA sequencing was performed on RNA extracted from 58 snap frozen incident bladder cancers (urothelial carcinomas) using RNeasy mini kits (Qiagen). Sequencing libraries were prepared from total RNA using Truseq Stranded Total RNA kits with Ribo-zero Gold ribosomal RNA depletion (Illumina) and paired-end sequenced using the Illumina NextSeq platform (2 × 75 bp). On average, 48 million reads were obtained per tumor sample.

### QC and Alignment

Raw fastq files were first processed using FastQC which makes diagnostic plots; subsequently, for each read, Trimmomatic (version 0.32) (22) was used to trim two bases from the start and clip where average phred score quality fell below 20. Quality-checked fastq reads were then mapped to the human genome (version GRCh37) and Ensembl gene annotation (release 87) using STAR aligner (ver 2.5.2b) (23).

### circRNA Prediction

Two different strategies were used to identify potential back-splicing events in the transcriptome which predict circular RNAs (circRNAs) to result in an *in-silico* reconstruction of circRNA coordinates. The CircExplorer2 algorithm (24) was used on the “chimeric.out.junction” file (from STAR aligner) containing information on potential non-linear alignments obtained for each sample. The second method, the DCC algorithm (25), differs from CircExplorer as it applies STAR separately on the R1 and R2 from the paired-end fastq data. To retain a high confidence data set, the output from each algorithm was filtered to select only those cases which had ≥4 reads supporting the back-splicing event in at least one of the samples.

Of the 4,571 circRNA candidates predicted by DCC, ~66% were also detected by CircExplorer2. Since DCC also provides count data for circRNAs and counterpart linear RNAs (host gene's mRNA) to permit calculation of circular-to-linear ratio, the output of the DCC algorithm was chosen for downstream analyses.

### Positional Bias of Exonic circRNAs

To identify any positional bias within the gene body, the genomic coordinates corresponding to RefSeq genes and their untranslated regions (UTRs) and coding sequences (CDS) were retrieved from UCSC Table Browser (hg19). In addition, bedtools (ver. 2.27.1) was used to overlap the exonic circRNA coordinates individually with the CDS, 5′ and 3′ UTR coordinates.

### Differential Expression

For the back-splice junctions implicated, read counts were resolved into circRNA and linear RNA (junction count) components using DCC. To control for different library sizes, circular-to-linear ratios were calculated after normalizing for RNA-seq library size resulting in counts per million (CPM); relative expression values were computed by log_2_ [(CPM(circularRNA)+1)/(CPM(linearRNA)+1)]. Circular-to-linear ratios were used to perform Analysis of Variance (ANOVA) using R (ver. 3.5.1) to identify tumor grade discriminating circRNA candidates (at adjusted *p*-value 0.05).

### Pathway Analysis

GO enrichment was carried out between a list of differentially expressed genes and the background of all parental genes of circRNAs using Gorilla (26). To compare sets of interests against the complete human background, we used the functional enrichment tool of the STRING database (version 11.0) (27). To further explore the biological processes affected by differentially expressed genes, we carried out pathway analyses using the Enrichr online tool (28). Pathways were adjudged using the KEGG (Kyoto Encyclopedia of Genes and Genomes) database with a *p*-value threshold of < 0.05.

In order to investigate whether the circRNAs showed different behavior compared to host genes in selected pathways, we selected previously-reported important bladder cancer pathways (29, 30). These include the pathways “DNA repair,” ERBB signaling, PI3K/AKT signaling, WNT signaling, EGFR signaling, MAPK pathway, and “chromatin remodeling,” as defined in the Signatures Database (MSigDB) (ver.7). In order to visualize the relationship of circRNAs to their parental genes in these pathways, we investigated if circRNA events were detected for each pathway member; if not, genes were excluded from further analysis. For the selected genes in the respective pathways, gene expression counts and circRNA counts were tabulated in two separate matrices. These matrices were then individually processed for box plot using ggplot2. To further describe the relationship of gene vs. circRNA expression, we calculated Pearson correlation values between the expression levels of the parental gene to the expression level of the circular RNAs (after normalizing the read counts for the circRNAs by library size). In order to assess potential correlations of circRNA expression to the overall pathway activity, we computed pathway signature scores (using geometric mean over the individual gene expression values, adding a pseudo-count of the lowest expression value in the set). These pathway signature scores were then correlated to the expression of the individual circRNAs in the pathway (Log_2_ CPM with initial pseudo-count one) and to the relative expression (as used in the differential expression analysis).

### microRNA Binding Site Prediction

To identify microRNA (miRNA) binding sites within the predicted circRNAs, we first downloaded the predicted target sites of conserved miRNA families from TargetScan (release 7.2) (31). Of the 122,607 total target sites in the dataset, there were only 192 sites with length >8 bp and, hence, only sites of length 7/8 bp (99.84% of total set) were taken forward for further analysis. This filtered target dataset was then assessed for coordinate level overlap within the circRNA coordinates using bedtools (ver. 2.27.1).

### Comparison to Other Datasets

The full set of circRNAs (supported by at least two reads in at least two different samples) from the study of Okholm et al. was kindly provided by the authors. CircRNAs detected in urine by exome capture were downloaded from the supplement from Vo et al. (32) If necessary, circle coordinates were lifted over using a python script based on the python package pyliftover (version 0.4, https://pypi.org/project/pyliftover/) and slightly different coordinate notions (as start coordinate given by either 0, or +1) have been unified and entries mapped using an in-house perl script. In order to compare if the differentially expressed circRNAs identified show the same behavior between grades in the Okholm et al. data, we computed their relative frequency (occurrence in a grade grouping divided by the fraction of that grade in the dataset) in low-grade and high-grade samples from Okholm et al. (removing the 7 papillary urothelial neoplasm samples in the set). We computed the same frequencies in our dataset by combining G1 and G2 into a “low/intermediate grade” set, and keeping G3 as high grade. For both sets, circRNAs with occurrence in at least three samples were selected for analysis. We correlated these relative frequencies using Pearson correlation.

## Results

### Clinical Phenotype of NMIBC Patient Samples

Our study cohort includes 52 NMIBC samples (grade1/G1 *n* = 17, grade 2/G2 *n* = 5, and grade 3/G3 *n* = 30) and 6 MIBC samples (all G3). The median age of the patients was 71 years and the male:female ratio was 6:1. There was no statistically significant difference in age distribution between the genders (Mann-Whitney *p*-value 0.26).

### Identification of Circular RNAs

**Figure 1:**
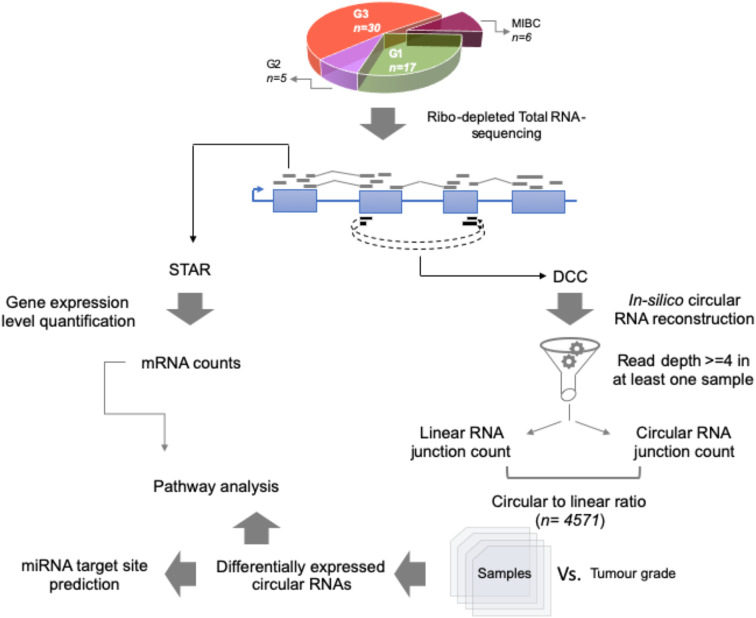
Work flow for identification of circular RNA candidates in context of non-muscle-invasive bladder cancer subtypes. Using 58 patient tumor tissue samples across G1, G2, and G3 tumor grades for NMIBC and additionally 6 MIBC samples, total RNA-sequencing was performed using Illumina platform. Coordinate boundaries for circular RNA candidates were *in-silico* reconstructed using DCC algorithm. For each circular RNA candidate, simultaneous evidence for back-spicing and linear splicing (circular RNA count and linear RNA count, respectively) were quantified which were then used to calculate the circular-to-linear ratio for each candidate. These ratios were then subjected to differential expression statistics using the limma BioConductor package. The differentially expressed circular RNAs across the tumor grade subtypes were then used for pathway analysis based on the host gene name. Further the circular RNA sequence body was checked for harboring potential miRNA target sites using TargetScan database.

For the total RNA sequencing of all 58 tumor samples, an average of 48 million reads per sample was obtained. To comprehensively identify circular RNA candidates and query the transcriptome status we used two strategies—parallel evaluation of the linear RNA and the circular RNA landscapes (Figure 1).

**Figure 2:**
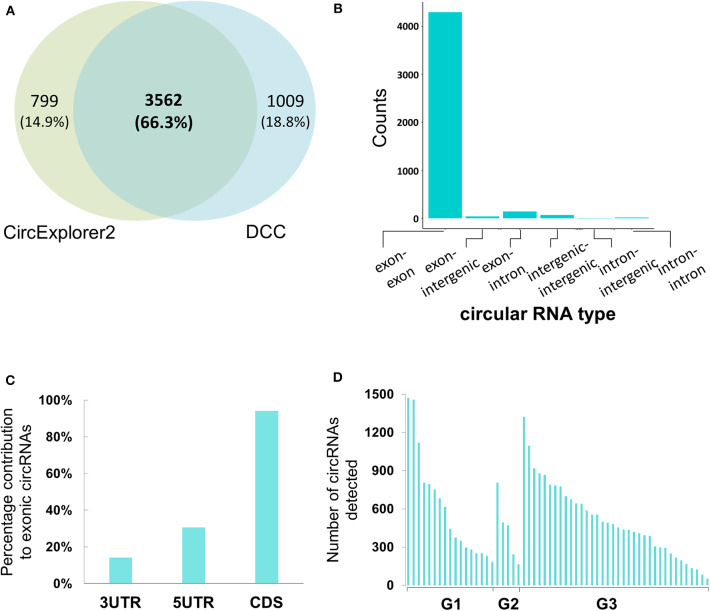
Genomic characterization of potential circular RNAs in Bladder cancer samples **(A)** Co-ordinate level overlap between circular RNA candidates detected by CircExplorer2 and DCC, respectively. **(B)** Types of genomic origin for circular RNA candidates. X-axis is showing the genomic origin pair across the back-splicing junction. Y-axis is the count of circular RNAs obtained from that pair type. **(C)** Within mRNA positional bias for circular RNA candidates. X-axis represents the three classes of space within mature mRNA body. Y-axis is the percentage contribution of each space type to exonic circular RNA candidates. **(D)** Tumor-grade wise distribution of circular RNA candidates. X-axis represents the 58 tumor samples categorized by the tumor grades of G1, G2, and G3, respectively. Y-axis represents the number of circular RNAs detected with more than 4 back spliced reads in the tumor sample.

For the circular RNA landscape, two different algorithms, CircExplorer2 and DCC, were used. While individually the two algorithms detected 4,361 and 4,571 candidates, respectively, there was significant overlap in the candidates identified (Figure 2A). In addition to the junction count for back-splicing events, the output from the DCC algorithm also provided the paired count for the linear events which allowed comparative evaluation; hence, for downstream analyses, results from the DCC were carried forward.

### Genomic Characterization of Circular RNAs

When reconstructing the boundaries of the circRNA candidates, the DCC algorithm annotates them on the basis of their genomic context (exonic, intronic, and/or intergenic); amongst all the circRNAs thus annotated, the exonic circRNAs were predominant (~94%) (Figure 2B). We then assessed positional bias (within the mRNA body) of the exons involved in circRNA formation. On further resolution of exonic circRNAs over the mature RNA sections of either the untranslated region (UTR; 5′ or 3′) and coding sequence (CDS), 94.1% of the exonic circRNAs were found to be within or overlapping the CDS; the values for those within or overlapping 5′ and 3′ UTR were 30.5 and 14.1%, respectively (Figure 2C). In addition to these, we looked at the tumor grades for presence of more number of circular RNA candidates in one grade vs. the other. We find that the median number of circular RNAs detected when seen grade-wise are 444, 471, and 468.5 for G1, G2, and G3 respectively (Figure 2D). Hence there didn't appear to be any strong bias in the distribution of circRNAs across the tumor grades.

### Gene Expression Differences Delineating NMIBC Tumor Grades

Gene level counts estimated from the total RNA-sequencing of the 58 tumor samples were used to identify genes with differentially expressed patterns between tumor grades. A total of 1,071 genes were identified at *p*-value threshold of 0.05 (after multiple-testing correction). Functional annotation of the gene set was performed and the pathways found to be significant were predominantly DNA-repair and cell cycle related.

### Identification of Dysregulated Circular RNA Between Tumor Grades

**Figure 3:**
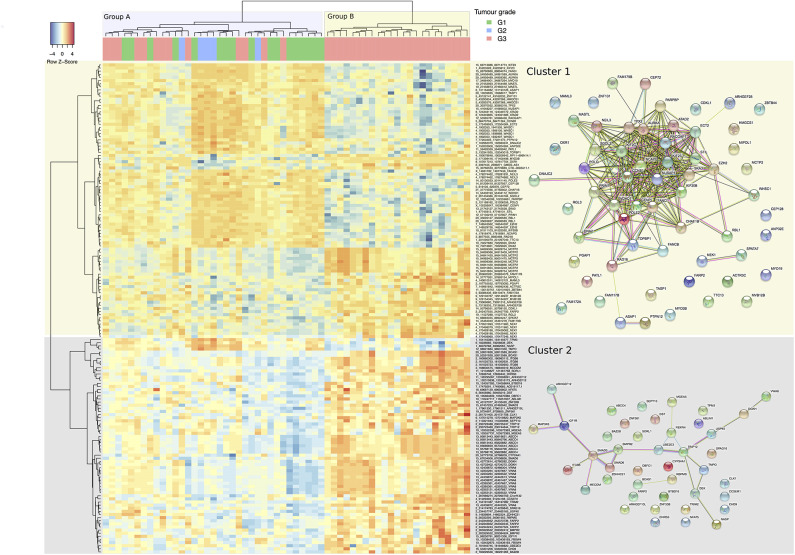
Heatmap representation of hierarchical clustering for 157 differentially expressed circular RNAs (Y-axis) by the 58 tumor samples across G1, G2, and G3 tumor grades (X-axis). Each cell value is the circular-to-linear ratio of an individual circRNA in a given tumor sample. The two main groups of samples branching out in the cluster representation are discussed in the text are color coded in the x-axis (Group A and B), and the two main clusters of circRNAs (Cluster 1 and 2) on the y-Axis. On the right, STRING sub-networks are pictured to illustrate functional connectivity between the parental genes of the circles. Individual functional enrichments of these groups are listed in Supplementary Table 1.

Using the DCC algorithm at the confidence threshold of ≥4 supporting reads in at least one sample, 4,571 circRNA candidates were detected. These candidates came from 2,430 unique genes with a ratio of 1.88 circRNAs per gene. The ANOVA analysis, based on relative expression, identified 157 circRNA candidates from 107 unique host genes as having significantly different levels of expression between the tumor grades (set “differentially relative expression of circular RNAs,” Supplementary Table 1). The parental gene list is functionally enriched in processes such as cell-cycle, DNA-repair, and cytokinesis when compared to the background of all parental genes of circRNAs detected (as reported by a GO enrichment analysis) (Supplementary Table 1). A heatmap based on these discriminative circRNAs is shown in (Figure 3), indicating their discriminative potential between tumor grades: the samples were grouped by the clustering into two main subgroups (denoted groups A and B in (Figure 3), separated by the initial branch, at the top of the clustering tree, on the X-axis). The first group comprises an inhomogeneous sub-cluster (A) with a mix of different grades, whereas the second main group (B) is homogenous for samples with grade 3. On the Y-axis, the circular RNAs are grouped by the clustering into two main clusters (Cluster 1 and 2). Both clusters exhibit sub-clusters with distinct expression patterns. In Cluster 1, for example, there are two sub-clusters with low expression of circRNAs predominant in the Group B samples, but rather upregulated in most of the Group A ones (mostly in the upper area of the heatmap). The parental genes of these circRNAs are enriched with functional processes relating to DNA replication, cell-cycle, and DNA repair (Supplementary Table 1), and reveal a highly connected module in terms of functional interactions (right panel of STRING interactions in Figure 3). This pattern is inverted in Cluster 2, for which Group B shows mostly high expression. However, the parental genes of circRNAs in this cluster do not exhibit a large amount of functional connectivity. Gene Ontology enrichment (albeit not significant after correction for multiple testing) for this cluster suggests involvement of circRNAs in regulative processes including SMAD3 and 6 (indicating a potential connection to TGF-b and BMP signaling) and Insulin-Like Growth Factor 1 Receptor, implying a potential role of these circular RNAs in facilitating progression and evasion of apoptosis (Supplementary Table 1), nevertheless, a functional interpretation remains difficult. Compared with a clustering based upon significantly differentially expressed genes (Supplementary Figure 1), the clustering appears of similar if not better discrimination between grades: as mentioned, Group B comprises Grade 3 tumors only with a distinct expression pattern of circRNAs. A separate but large fraction of Grade 3 tumors are clustered together with Grade 1 and Grade 2 samples in Group A. Grade 2 exhibits a tendency to cluster together in Group A which shows high expression of a small sub-set of circular RNAs (with parental genes TPM3, DEK, NASP, and TMPO) and which is low for most members of Group B.

We also collected circRNA candidates whose parental genes are affected by NMIBC grade as detected by differential expression analysis on the gene level: the set of all genes with any circRNA detected (*n* = 2,430) was overlapped with the set of differentially expressed genes (*n* = 1,071; Supplementary Figure 1). This resulted in an intersection of 143 host genes that are differentially expressed between tumor grades and exhibit circularization (set “gene differentially expressed at mRNA level,” Supplementary Table 1). Finally, genes where the circRNAs are differentially expressed with little influence of the host gene expression dynamic between the different grades were identified by subtracting the “circles in differentially expressed genes (*n* = 107)” set from the “differentially relative expression of circular RNAs” set (*n* = 143): 24 such circRNAs were identified (Supplementary Table 1). These 24 circRNAs did not exhibit functional enrichment.

### Pathway Analysis

Visual inspection of the expression levels of linear RNA and circRNA (gene level summarized) for genes with circRNA in bladder cancer relevant pathways (Supplementary Figure 2), indicates non-regular patterns of circRNA expression which do not follow obvious trends in relation to the linear form. Whereas, for the linear RNA the spread of expression level per gene tended to be more tightly bound, the circRNA spread was much more variable across genes, samples and tumor grades. In concordance, most circRNA expression levels show only poor correlation to the expression of the parental gene (Supplementary Table 3). In general, the expression of individual circRNAs within these pathways does not correlate with the overall pathway activity (Supplementary Table 2). This is also the case when correlating the relative circular expression with the pathway activity. However, more significantly correlated instances can be detected in the latter analysis (at a *P*-value cut-off at 0.05: 66 for the relative vs. 174 for the overall expression, Supplementary Table 2), for example, a circRNA originating from FANCI, a gene involved in DNA repair, shows a strong negative correlation with the DNA repair process (*R* = −0.85, *P* < 2.2^−16^).

### Frequency of MicroRNA (miRNA) Binding Sites Within circRNAs

Using the TargetScan dataset of predicted miRNA target sites, a total of 132 circRNAs (hosted by 99 unique genes) were found to harbor target sites for 141 miRNAs. Amongst the host genes whose circRNAs harbor miRNA target sites, 4 were differentially expressed (both at the mRNA and at circRNA level) between the tumor grade subtypes: *CDKL1, HP1BP3, MVB12B*, and *TMPO*. The circRNAs from these 4 host genes harbor target sites for 11 different miRNAs: miR-7-5p, miR-30-5p, miR-31-5p, miR-96-5p/1271-5p, miR-139-5p, miR-181-5p, miR-182-5p, miR-433-3p, miR-489-3p, miR-493-5p, and miR-543. The 5 most recurrent miRNAs with target sites within circRNAs are the miR-15/107 family, miR-101-3p, miR-204-5p/211-5p, and miR-203a-3p. Many of these miRNAs have been reported to be implicated in tumor biology and/or be predictive of response to drug treatment (33–38). The results are summarized in (Supplementary Table 1).

### Comparison to Other Datasets

The comparison between our dataset and that of Okholm et al. identified 3,361 overlapping circRNAs [77% from this study, 22% of the 15,223 instances in the data of Okholm et al. (21)] (all instances listed in Supplementary Table 4). For the circRNAs significantly differentially expressed between grades, 108 could be found in both datasets (69%). To each of these 108 circRNAs we assigned their relative frequency to occur in the set of low/medium and high-grade tumors, respectively, to describe their tendency to be grade specific. We compared these between the two datasets and found a strong correlation (*R* = 0.47, *p* = 2.3e−07; Supplementary Figure 3), indicating that their grade-specific behavior can be detected independent of the cohort. Notably, the identified grade-stratifying candidate circRNAs do not include the two proposed progression biomarkers identified by Okholm et al., circHIPK3 and circCDYL.

We identified 552 of our 4,116 circRNAs (12%) in the urine dataset from 13 prostate cancer patients provided by Vo et al. (32). This urine dataset comprises 1,092 circRNAs, resulting in a 50% recall of the urinary circRNAs in at least one of our samples. Within the set of significant differentially expressed genes (*n* = 157), 8% (13 circRNAs) can be found in the urine dataset (Supplementary Table 4).

## Discussion

In this work we have investigated circRNAs from a cohort of NMIBC patients. The general genomic properties of the circRNAs are in keeping with earlier findings in other studies: circRNAs are mostly connecting exons, and are mostly detected within coding sequences. The total number detected varied between tumors and was lowest in grade 2 tumors (although only 5 grade 2 tumors were analyzed). However, detected number of individual circRNAs was always >100 and up to >1,000, providing a considerable dataset for further investigations. Since our dataset is well-annotated by tumor grade (G1, G2, and G3), we aimed to delineate a subset that stratified the dataset by grade using a supervised approach. This approach selected 157 circRNAs, and cluster analysis resulted in distinct subgroups of tumors (Figure 3). The clustering presented here is a visualization of the results of the differential relative circular expression analysis. The resulting heatmap might slightly vary by choice of parameters and clustering algorithms; however, the result implies the existence of molecular subtypes in terms of circRNA expression, with varying tendencies of certain grades to exhibit the subtype.

Gene expression differences among NMIBCs have been reported previously, and pathways relevant to tumor biology in bladder cancer are ERBB2, PI3K-AKT, cell cycle, MAPK, and DNA-damage repair. One of the primary questions driving investigation into any form of cis-regulatory RNA is how it affects the expression level of the host gene. In this regard, we investigated the comparative expression levels of the linear and circular RNA for selected pathways. We observe that, for a given pathway, the genes (linear RNA) retain a similar trend of expression pattern between tumor grades, whereas the circRNA levels are much more variable. This could be an effect of increased variability in the circRNA count data since the majority are at low expression levels compared to linear RNA. Nonetheless, for a particular grade group, we observe a discernible shift in the expression pattern of some genes. In the ERBB2 pathway, the *ERBB2* gene is consistently the highest expressing linear gene among all the three tumor grades but, for circRNA, *SOS2* has the highest expression across the grades. Similarly, in the PI3K-AKT pathway, *ACTR3, SMAD2, STAT2*, and *ACACA* are most highly expressed in linear RNA, whereas for circRNA, *MAPK8* appears to have higher expression. These observations indicate that within the same pathway there are likely different regulatory processes acting on linear vs. circular RNA. This is also indicated by the lack of correlation between the expression levels of circRNAs to their parental genes in the investigated pathways.

We also found that the activation of a pathway (as measured by gene signature score) is mostly independent of the circRNAs within the pathway, but less so when looking at relative expression. These instances, for which the relative expression either positively or negatively correlates with the pathway expression, might be under certain biological constraints. In the case of a negative correlation, the cancer cell seems to avoid upregulation of circRNA and the observation could be explained by a steady level of circRNAs with increasing pathway activity. With a positive correlation there may be a cis-acting role on the pathways, since their relative expression increases with pathway activity but is not bound to the expression of the parental gene. The way by which these circRNAs potentially interact with pathways of interest cannot be directly deduced from this analysis. However, systematic correlation studies may help to identify candidates for functional follow up screens.

Interestingly, four predicted miRNA sponges could be found differentially expressed. We found cancer-related pathway-specific genes being targets of the top recurrent miRNAs (targeting circRNA). The miR-15/107 family is found to have target sites within circRNAs from host genes *CDC42, MGEA5, CHPT1, GPATCH2L, TSEN2, RPL14, PLD1, TFRC, PDLIM5*, and *PTK2*. The genes *CDC42, PLD1*, and *PTK2* are involved in the EGFR signaling pathway; the miR-15/107 family is reported to have tumor suppressor properties (34, 39), and the circRNAs from the above genes for EGFR signaling pathway can act as “sponges” to exert oncogenic effect in the context of NMIBC. Similarly, miR-204-5p/211-5p has targets within *CDC42, KHDRBS1, ASH1L, MDM2, GPATCH2L, WSB1, ZNF638*, and *AGTPBP1* genes. Along with *CDC42, MDM2* is involved in DNA damage responses. Additionally, the duo of miR-204-5p/211-5p has recently been reported to be involved in resistance to BRAF inhibition (40). Hence, circRNAs harboring target sites for miR-204-5p/211-5p can have important implications for tumor treatment and progression (41, 42).

The number of samples in our study is still small, especially for Grade 1 and Grade 2 samples and the reported list of differentially expressed circRNAs provides a list of interesting candidates that can be tested in future studies. However, the existence of another bladder cancer cohort suitable for circRNA detection by Okholm et al. gave us the opportunity to compare two different cohorts and to test our findings in their data. The grade-specific relationships of circRNAs could be identified in the dataset of Okholm et al. (21). This indicates a certain amount of transferability between independent cohorts, despite different computational pipelines, and provides some mutual validation of the two cohorts. The urinary circRNA in the dataset from prostate cancer patients provided by Vo et al. would be expected to comprise both prostate cancer specific circRNAs and circular RNAs from normal bladder tissue, and was therefore suitable to investigate whether circRNAs detected in our study can be detected in urine. Indeed, we found 50% of the circular RNAs in the urine set in at least one of our bladder tumor samples, and 12% of the bladder tumor circRNAs from this study exist in the urine dataset. It is therefore likely that a high proportion of circRNAs expressed by bladder tumors will be detectible in urine, notwithstanding the circRNAs differentiating between grades are slightly under-represented with 8% recall. The latter observation might indicate that the discriminative set comprises instances that are bladder cancer specific; however, given the relatively small size of this dataset, the significance of this finding remains unclear. Nevertheless, these observations indicate a need to further investigate the potential of urinary circRNAs as diagnostic and/or prognostic biomarkers.

## Conclusion

The present study provides a step in the comprehensive evaluation of circRNAs in the context of bladder cancer. Intriguingly, despite their potential function as microRNA sponges, circRNAs are potentially affecting host mRNA levels at the transcriptional stage, as compared to post-transcriptional control by miRNAs. We have also identified circRNA candidates worthy of further functional investigation, and comprising potential miRNA sponges and circRNAs correlated to pathway expression. Our analysis indicates circRNA differences between bladder cancer grades, and their relative expression levels may provide an additional modality for patient risk stratification. Furthermore, since circRNAs have a longer half-life in the extracellular milieu than other RNAs and are detectable in urine, they may be useful non-invasive biomarkers for bladder cancer diagnosis and risk stratification.

## Data Availability Statement

RNA sequencing data for the bladder cancer patients have been submitted at the European Genome-Phenome Archive (EGA) (https://ega-archive.org/) under accession code: EGAS00001004358.

## Author Contributions

AG, RA, and RB conceived and designed the study. NG and DW provided technical and material support. BA, MZ, KC, NJ, and RB have been involved in acquisition of funding, samples, and data. AG and RA analyzed the data, AG, RA, DW, and RB wrote and revised the manuscript. All authors read and approved the manuscript.

## Conflict of Interest

RB has contributed to advisory boards for Olympus Medical Systems and Janssen. NJ has contributed to advisory boards for Merck USA and Pierre Fabre. The remaining authors declare that the research was conducted in the absence of any commercial or financial relationships that could be construed as a potential conflict of interest. The handling Editor declared a shared affiliation, though no other collaboration, with several of the authors RA, AG, DW, NG, BA, MZ, KC, NJ, and RB.
