# Supplementary material for: Back-Splicing Transcript Isoforms (Circular RNAs) Affect Biologically Relevant Pathways and Offer an Additional Layer of Information to Stratify NMIBC Patients
Source: Front Oncol. 2020 May 22;10:812. doi: 10.3389/fonc.2020.00812 (PMC7326039; doi:10.3389/fonc.2020.00812)
Supplement: Supplementary file 5 [file Image_1.PDF]

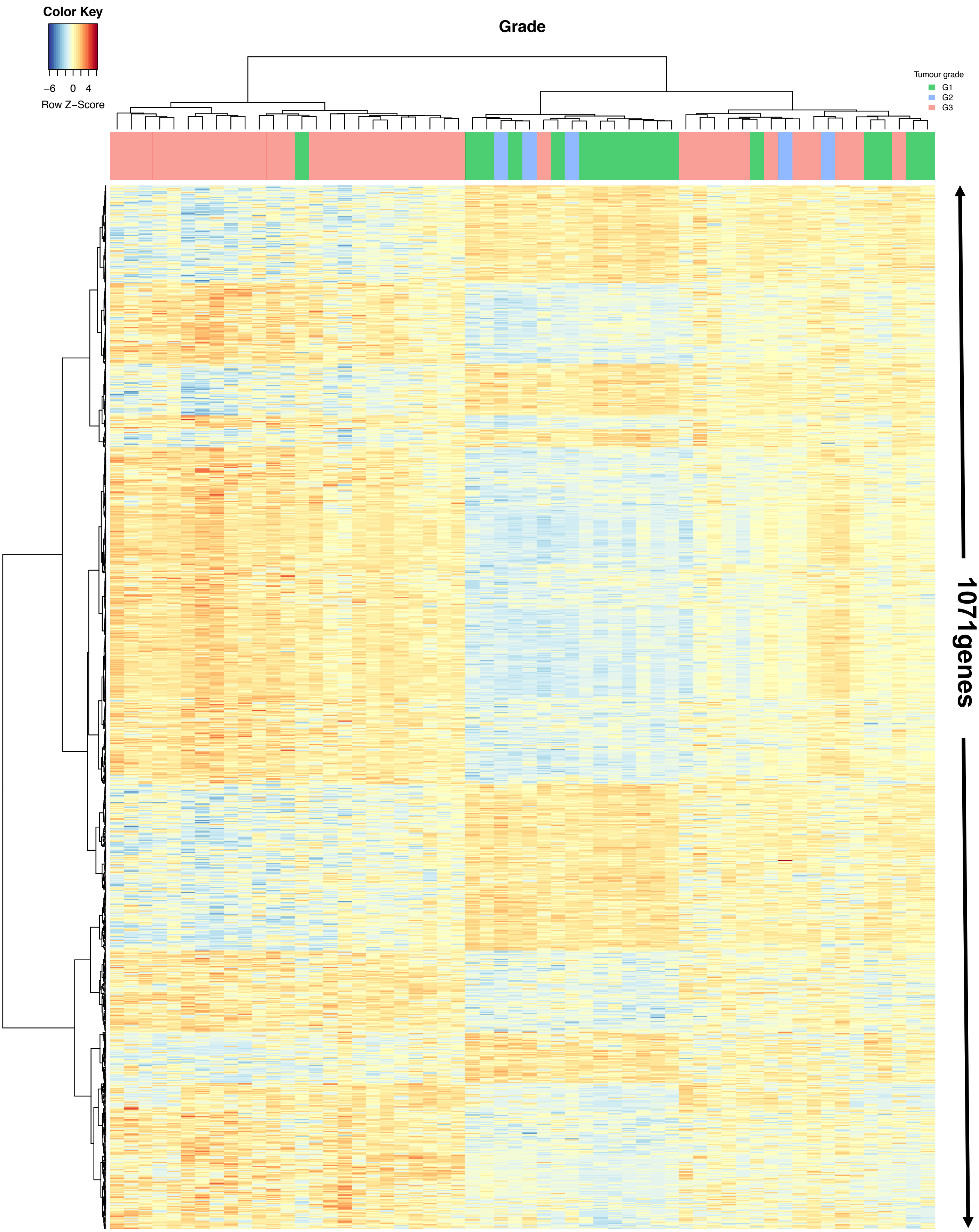

Supplementary Figure 1. Heatmap representation of hierarchical clustering using Ward.D2 method for 1071 differentially expressed genes (Y-axis) by the 58 tumour samples across G1, G2 and G3 tumour grades (X-axis). Each cell value is the normalized count of an individual gene in a given tumour sample.

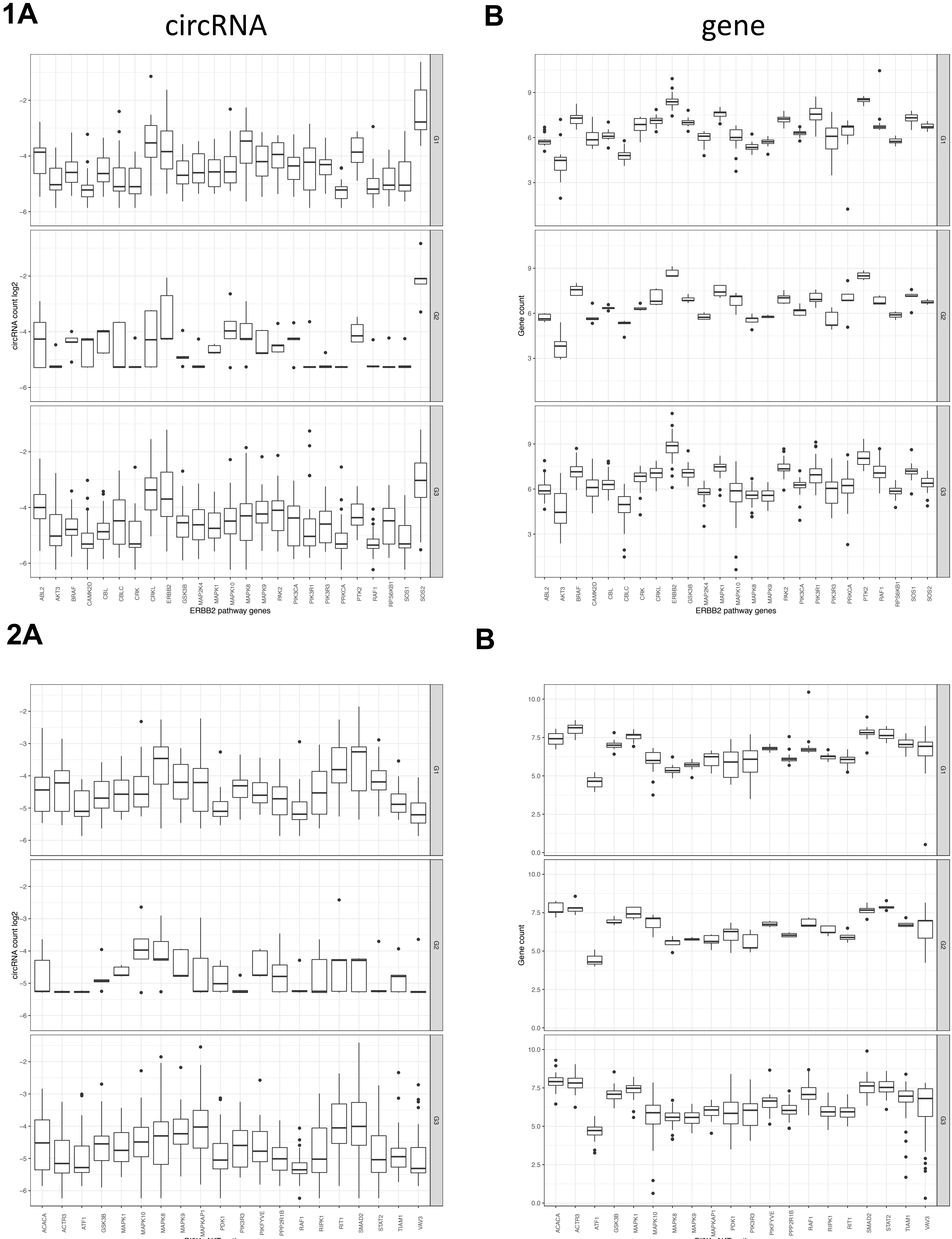

Supplementary Figure 2. Box plot of selected pathways (1. ERBB2, and 2. PI3K/AKT pathways respectively) for circular RNA expression in A and gene level expression in the B panel between G1, G2 and G3 stages. CircRNA count (left) computed as log2(CPM), gene count is TPM after voom normalization (right panel).

3A circRNA

B gene

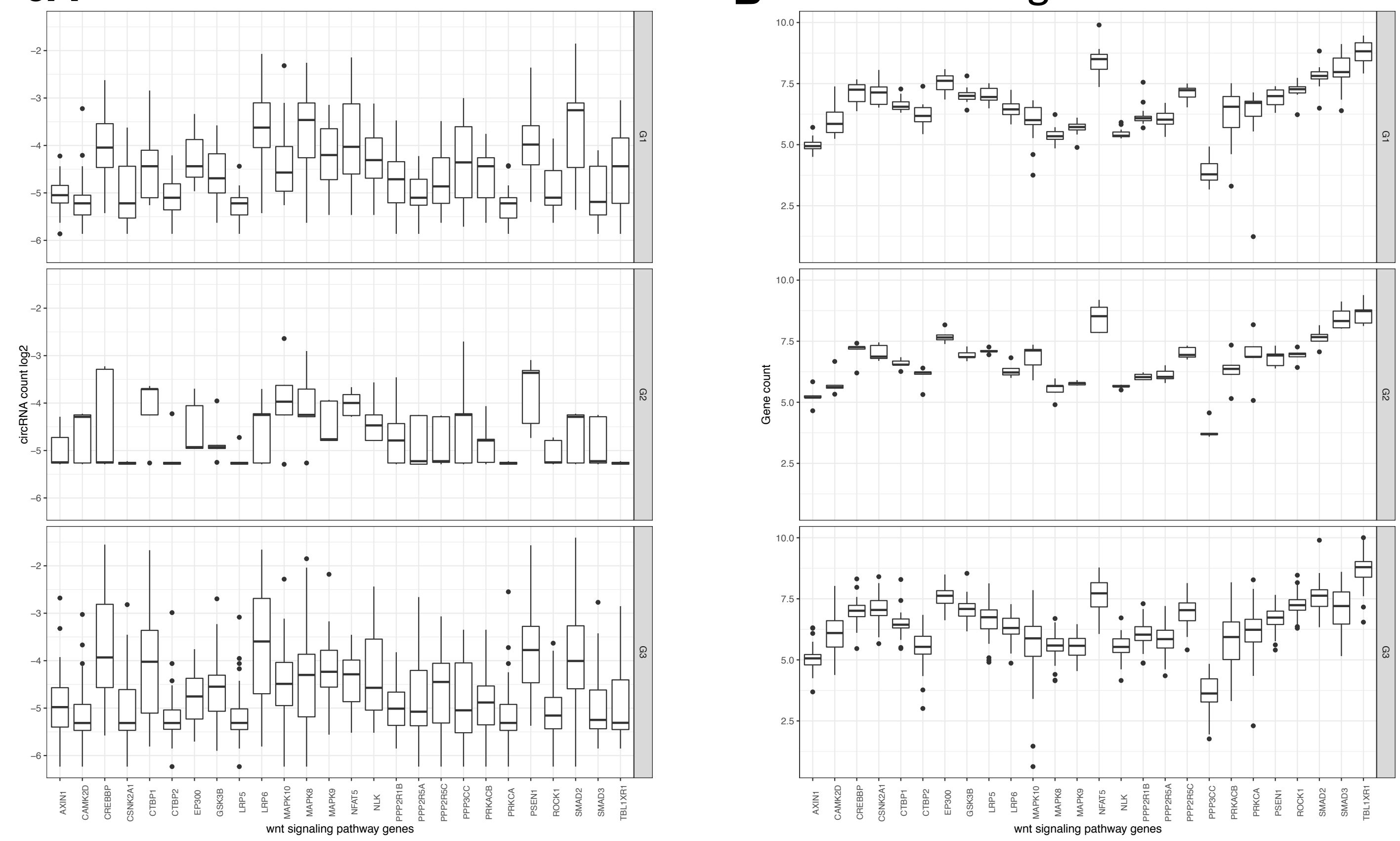

4A

B

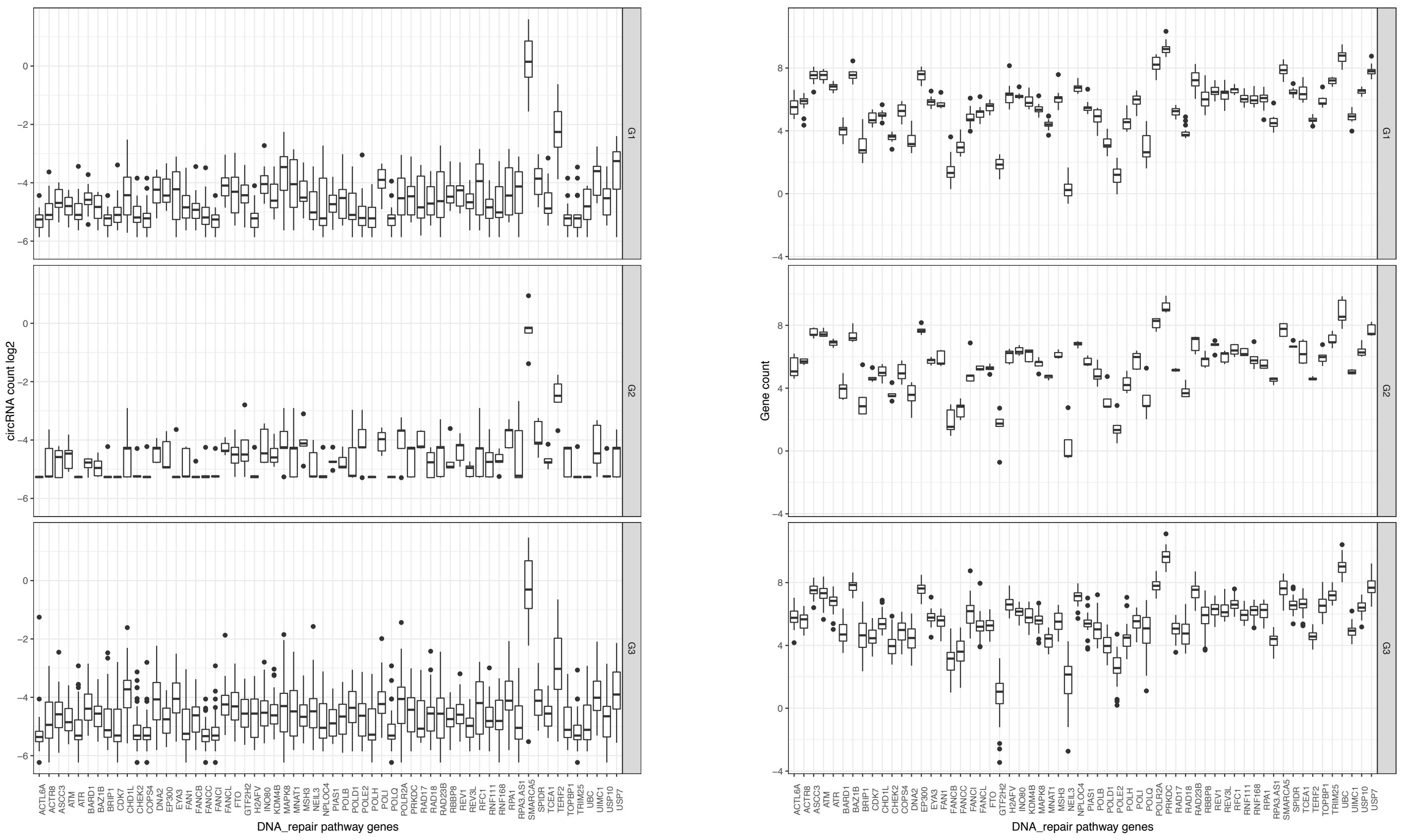

Supplementary Figure 2 Box plot of selected pathways (3. Wnt Signaling, and 4. DNA repair pathways respectively) for circular RNA expression in A and gene level expression in the B panel between G1, G2 and G3 stages. CircRNA count (left) computed as log2(CPM), gene count is TPM after voom normalization (right panel).

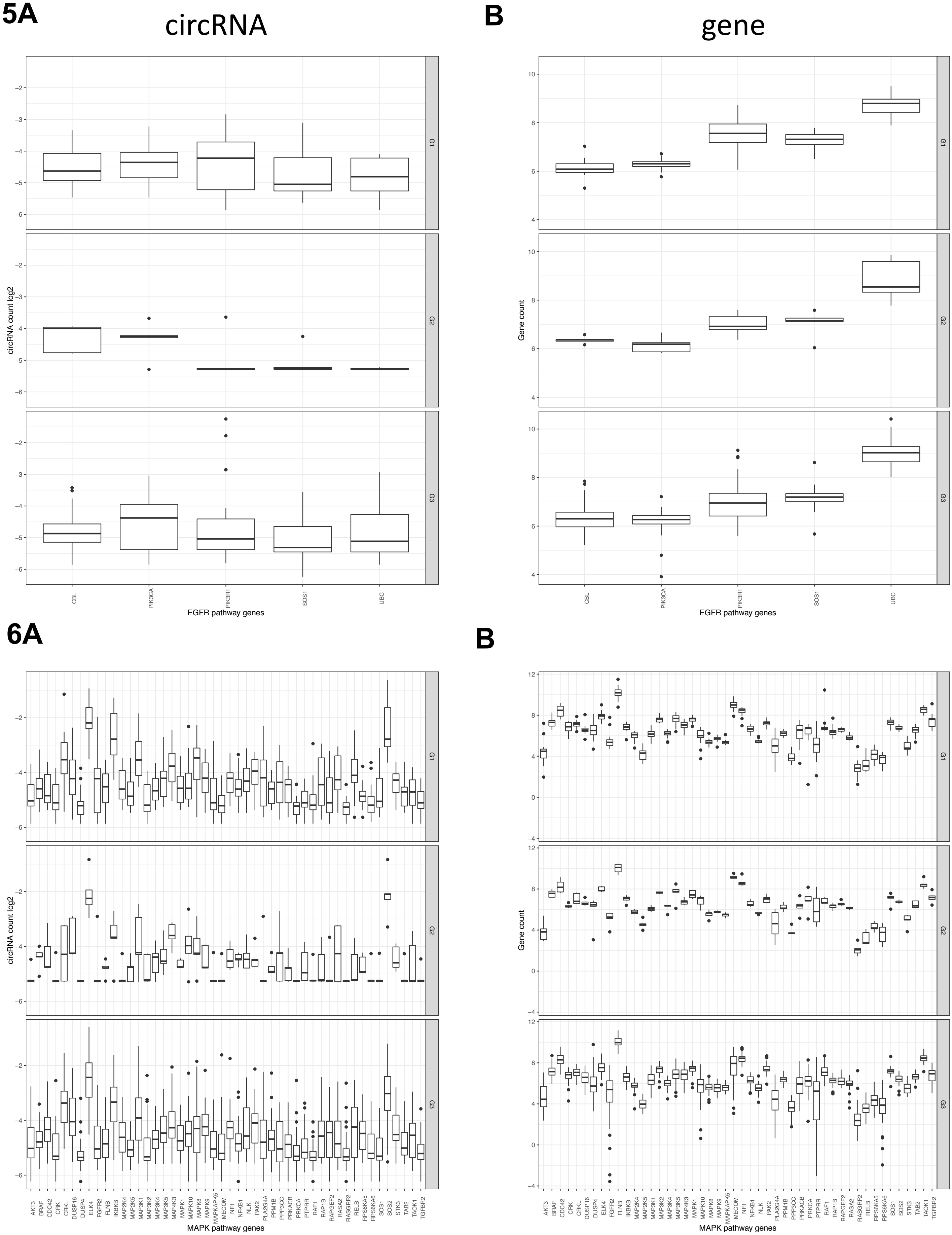

Supplementary Figure 2. Box plot of selected pathways (5. EGFR, and 6. MAP Kinase pathways respectively) for circular RNA expression in A and gene level expression in the B panel between G1, G2 and G3 stages. CircRNA count (left) computed as log2(CPM), gene count is TPM after voom normalization (right panel).

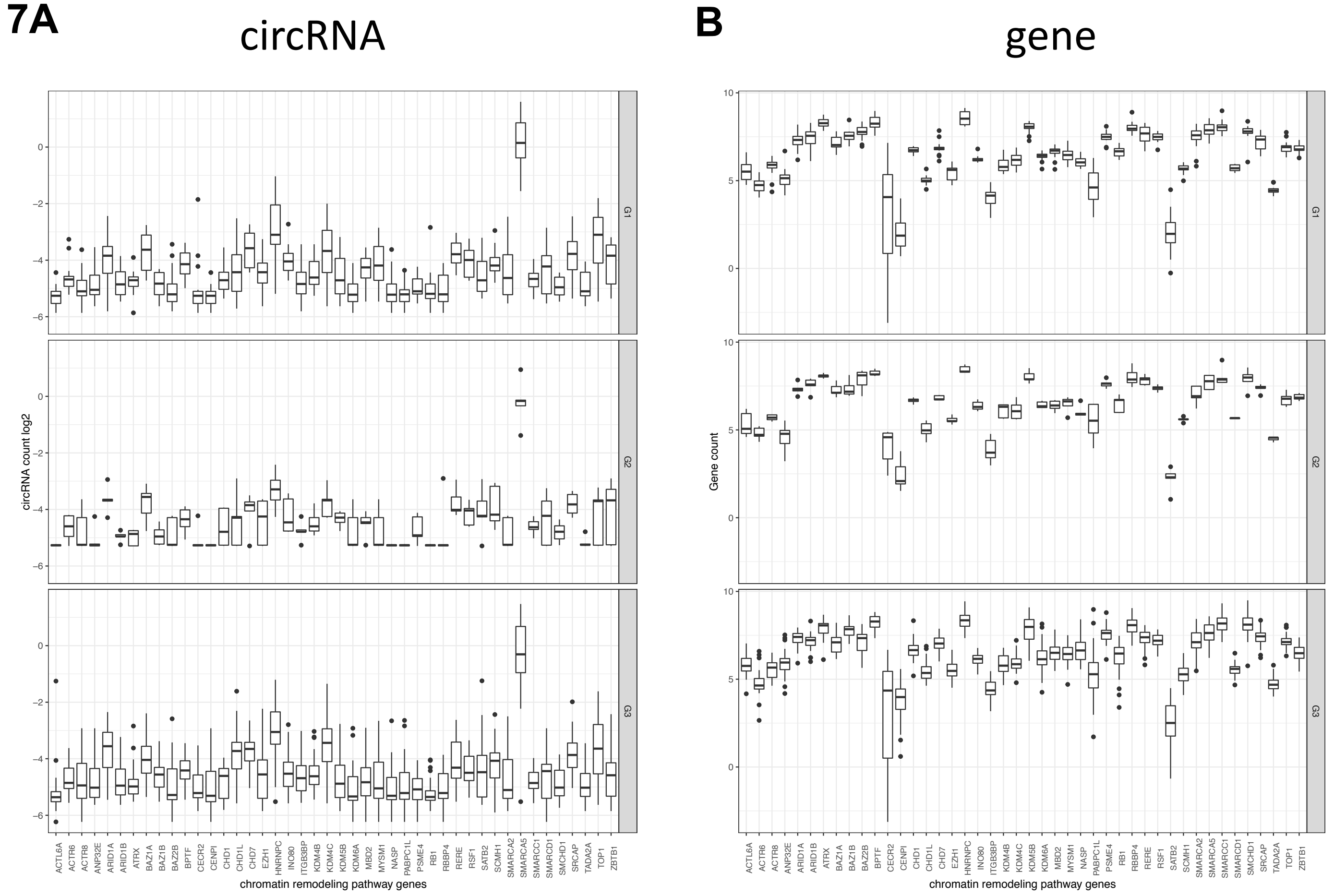

Supplementary Figure 2. Box plot of selected pathways (7. Chromatin remodeling) for circular RNA expression in A and gene level expression in the B panel between G1, G2 and G3 stages. CircRNA count (left) computed as log2(CPM), gene count is TPM after voom normalization (right panel).

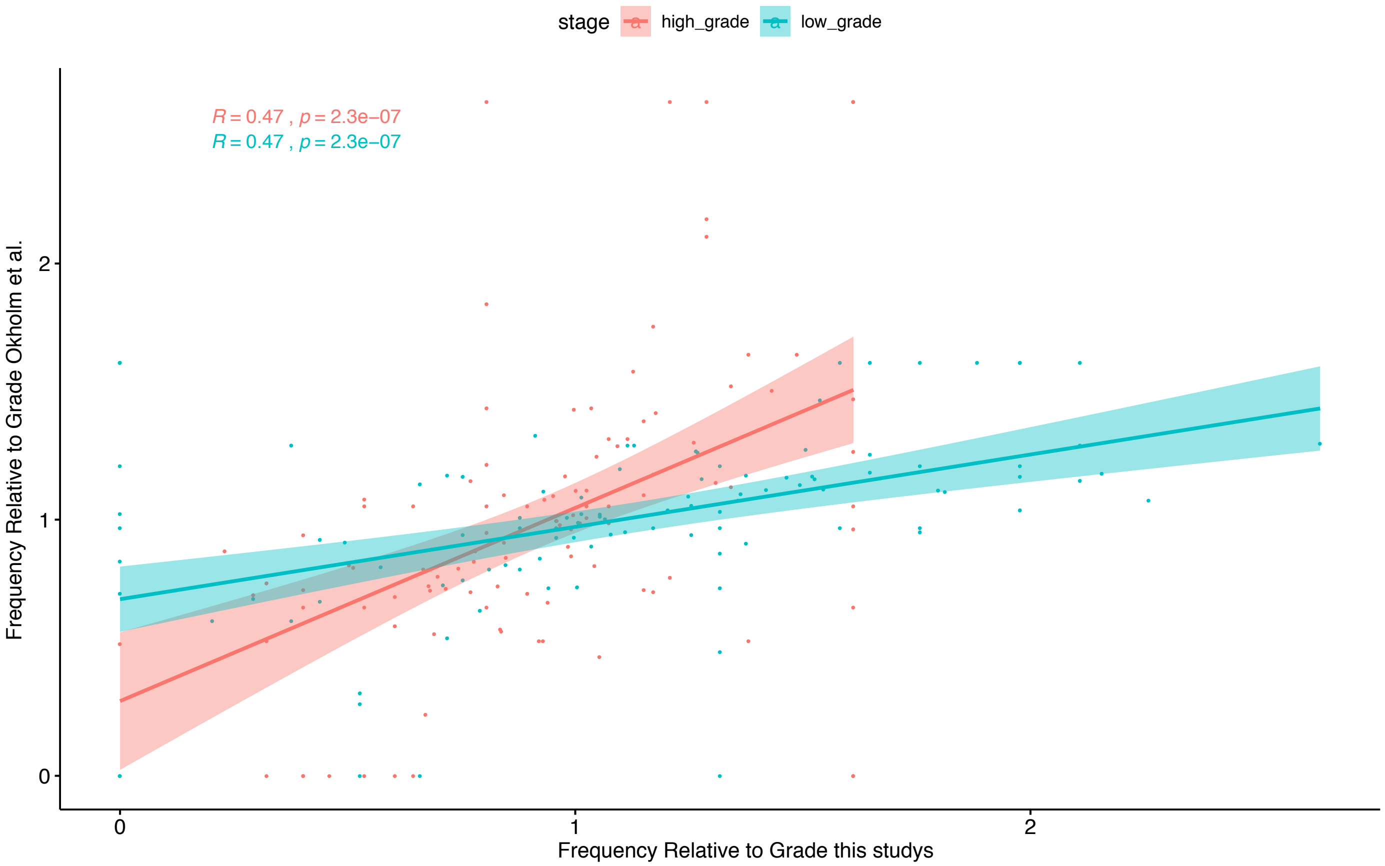

Supplementary Figure 3 . Correlation of relative frequencies between this study (x-axis), and the study of Okholm et al. 2017 (y-axis). The values represent the relative frequency in samples from either high grade (in this study: G3) or low grade (in this study: low and intermediate, G1 + G2) for the significant differentially expressed circular RNAs. Blue: low and intermediate grade set (G1+G2), red: high grade set (G3).
